# The NIH BRAIN Initiative’s Impacts in Systems and Computational Neuroscience, 2014–2023

**DOI:** 10.1101/2025.01.30.635684

**Published:** 2025-02-01

**Authors:** Farah Bader, Clayton Bingham, Karen K. David, Hermon Gebrehiwet, Crystal Lantz, Grace C.Y. Peng, Mauricio Rangel-Gomez, James Gnadt

**Affiliations:** 1 Program Officer,; 2 Program Analyst,; 3 BRAIN Team Co-Leads (current and emeritus),; 4 corresponding author

## Abstract

At the 10-year anniversary of the NIH BRAIN Initiative, this report analyzes the impact of the initiative’s functional neuroscience ecosystem as funding experiments in the domains of systems and integrative neuroscience, and computational neuroscience, with an eye on comparison with other funding models and best practices.

## Introduction:

The *Brain Research Through Advancing Innovative Neurotechnologies*^®^ Initiative (BRAIN Initiative) was launched in Fiscal Year 2014 from a 2013 U.S. Presidential directive, guided by evaluation from an advisory committee to the NIH Director. The advisory committee conveyed to NIH a mission concept in the Brain 2025: A Scientific Vision with seven priority areas (1. Discovering Diversity, 2. Maps at Multiple Scales, 3. The Brain in Action, 4. Demonstrating Causality, 5. Identifying Fundamental Principles, 6. Advance Human Neuroscience, 7. From the BRAIN Initiative to the Brain). A special Congressional appropriation for the BRAIN Initiative provided funds to execute these priorities and allowed the BRAIN Initiative to focus on progressive and adventurous projects and programs outside of standard NIH Institute/Center (IC) constraints, and across IC missions.

The core philosophy of the BRAIN Initiative is to understand the brain as a complex system that gives rise to the diversity of functions that allow us to interact with, and adapt to, our physical and social environments. Such an understanding is necessary to promote brain health, and to prevent and treat neurobehavioral and neurological disorders (Miller et al., 2024). Like any complex system, one must understand how the brain works in order to know how to fix it when it dysfunctions. All brain disorders, including neurological, mental and behavioral conditions, manifest as system dysfunctions that must be understood as such for effective therapeutic cures and prevention. Thus, the NIH BRAIN Initiative has created a unique synergy with its partner NIH ICs that dramatically advances basic research on brain function, complementing the mission interests of the ICs in preventing and treating specific diseases.

Under the guidance of the Directors of 10 NIH ‘neuro’ ICs, Scientific Program Directors and Specialists with expertise in various approaches that matched the advisory committee guidance, convened to interpret and implement the mission concepts of the BRAIN 2025 report. The systems neuroscience program - after splitting from the technology development component in FY2015 - was charged with implementing the investigative, functional neuroscience of the mission directives, spanning all 7 priority areas, as noted above. Non-invasive approaches in functional human neuroscience were pursued as a separate track focused on neuroimaging technologies across scales.

The Integrative and Quantitative Approaches Team was convened to design funding programs that would address mechanisms of how the nervous system works as an integrated system, or ‘heksor’ (Wolpaw and Kamesar, 2022). Strategically, this fundamental, systems neuroscience component focused on combining *in vivo* behavior, dynamic neural systems and computational neuroscience approaches (BRAIN 2025). Since most functional systems of the nervous system are mediated by dynamic neural circuits, this component of the BRAIN Initiative is sometimes referred to as the ‘BRAIN Circuits Program’. However, flexibility was built into the funding programs to encourage understanding of non-neuronal systems influence on dynamic circuits (Bader et al., 2024), and to allow studies of *in vivo* ‘behavior’ of well-defined neural systems within the nervous system.

## The NIH BRAIN Circuit Programs

**Figure 1:**
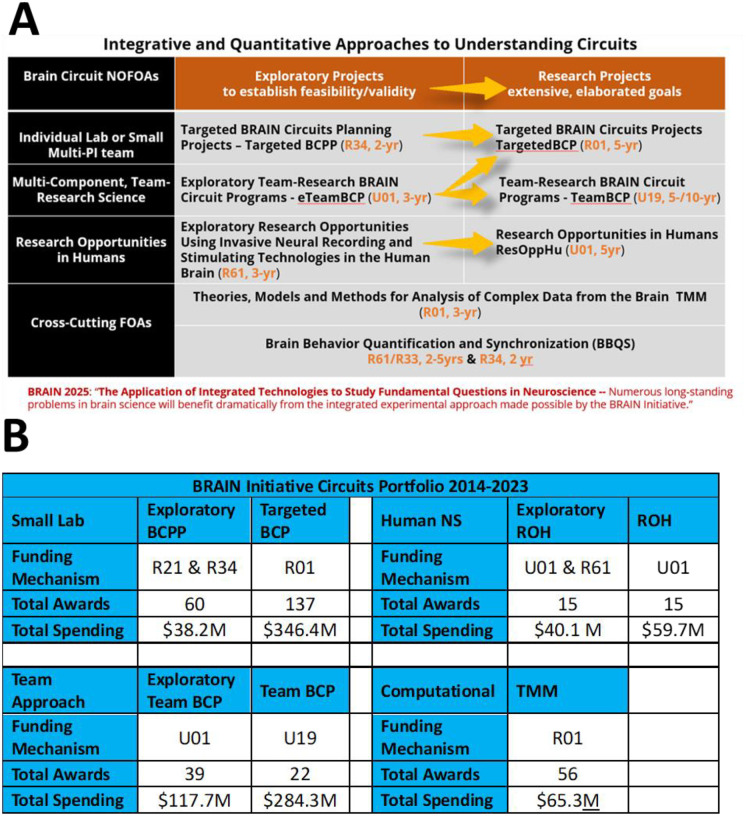
A. Ecosystem of funding mechanisms. Exploratory versions of each research track are designed to provide an enabling step, where needed, that goes through peer-review to larger, more elaborated awards. B. Tabulation of number of awards and total spending for 2014–2023, not including approved out years of funds yet to be expended.

From the beginning, this functional neuroscience approach adopted a set of guiding scientific principles that defined the priorities and context for developing the content of the ‘circuit-busting’ funding announcements (David et al., 2020). Based on these principles, the BRAIN Circuits Program created an ecosystem of funding mechanisms illustrated in Figure 1A. Description of activity codes for award mechanisms discussed below can be found on the NIH Grants & Funding website. The general format of this integrated approaches program has been to have 2-/3-year exploratory forms of funding, followed by a peer-reviewed opportunity to compete for an expanded 5-year award, in each emphasis area. Note that the exploratory projects are not a requirement of the expanded awards but offer an enabling, or pilot, step where useful. Research topics were prioritized to be investigator-initiated concepts in fundamental systems neuroscience in three major research tracks: 1) targeted, single-lab-sized or limited multi-PI research proposals, 2) team-research approaches that could only be successful as integrated approaches across biological scales, disciplines and/or species, 3) human-neuroscience research opportunities afforded by direct, intracranial access to recording and manipulating in the human brain. This format emphasizes peer-reviewed, investigator-initiated selection of scientific merit. Specific analysis of the multi-component, team-research programs is presented elsewhere (Bader et al., 2024). Specific analysis of the investigative Research Opportunities in Humans program (ROH) is planned as a separate publication.

Awards in these BRAIN Circuits Programs were required to include multi-scale approaches combining *in vivo* behavior, dynamic neural systems and quantitative methods. Figure S1A shows a word cloud made of the unique, competing BRAIN Circuits Program award titles and abstracts which illustrates that the salient research topics from this PI-initiated program reflect the research emphases in fundamental behavioral, systems and computational neuroscience. The funding mechanisms, number of awards and total committed budget through 2023, per funding RFA, are listed in Figure 1B. This total 10-year expenditure in quantitative systems neuroscience has invested approximately $1B into fundamental neuroscience research. Note that, as directed by the BRAIN 2025 report, the BRAIN Circuits Program balanced funding between small-lab and collective team research in the Targeted and Team track at $384M vs. $403M, respectively. The human ROH program composed about 10% of the total budget at $100M.

As part of the functional neuroscience approach, there was also a strong desire to promote better developed theories into fundamental systems neuroscience. Taking into account that all the BRAIN Circuits Program funding mechanisms were required to include quantitative methods, and to not replicate or compete with the highly successful NSF/NIH Collaborative Research in Computational Neuroscience, we crafted a ‘tool building’ program in computational neuroscience with an emphasis to develop and disseminate computational tools in the form of novel Theories, mechanistic Models, or mathematical Methods (TMM, 3-year R01). Parameters built into the program as requirements included 1) End user evaluation of the proposed tools, 2) Experimental studies limited to model parameter estimation and/or validity testing of the tools being delivered. In order to focus the applications on mechanistic understanding at the level of dynamic circuits, after the first cycle of applications an additional condition specified that products of the research must include knowledge at cellular and sub-second temporal resolution. The TMM 10-year award commitment to computational NS was 7% of the total expenditures at $65M.

The popularity of the top tool-products generated by TMM awardees can be quantified by Github stars (Table S2A), which indicate that a user likes or finds a software tool useful. Topics with high popularity include calcium imaging methods, multiple encoding/decoding tools, multiple model-building exercises and sophisticated statistical methods. The popularity of the top repositories from the TMM program can be quantified by Github stars and watchers (Table S2B). These two metrics can be reflective of active repository developers and contributors, which include multiple calcium imaging codes and a matrix factorization tool for imaging analysis.

In the interest of improving measures of high-content, high-temporal resolution of behavior to match that available for neural activity, a novel research track in Basic Behavioral Quantification and Synchronization (BBQS) was initiated in FY2023. There are staged funding tracts specifically for human behavior and for organismal behavior. The human clinical tract employs an administrative review for progression from early to elaborated stages (R61 to R33). Whereas, the organismal tract employs a peer-reviewed progression from enabling (R34) to elaborated stages for non-human (U01) and comparative human/non-human studies (U01). These programs are expected to better develop and engage dynamic behavioral and environmental assays integrated with dynamic measure of the neural systems of study. As a newly launched funding program, it is now premature to assess overall impacts for these programs in this report.

## 10-year Impact Evaluation

Now 10 years since conceiving this landmark initiative to better understand how the brain works, this report analyzes the impact of this functional neuroscience ecosystem as funding experiments in the domains of systems and integrative neuroscience, and computational neuroscience, with an eye on comparison with other funding models and best practices for how best to support fundamental, investigative neuroscience.

Figure 2A illustrates the annual NIH research investments committed in investigative neuroscience by year for the 10 years before and after the launch of the BRAIN Initiative. Using the NIH RCDC Terms coding (Research, Condition, and Disease Categorization), we found that “Neurosciences Research” best captured the BRAIN Circuits Program circuit-based research approaches with few false negatives. Thus, we constructed a timeline of NIH baseline funding of neuroscience research similar to the BRAIN Circuits Program portfolio using the RCDC terms for ‘neurosciences research’ [OR] ‘computational neuroscience’ (Sys&CompNS2004_2009, Sys&CompNS2010_2015, Sys&CompNS2016_2019, Sys&CompNS2020_2023), excluding BRAIN Initiative awards. This annualized baseline was tabulated from sums of Awarded Total Costs by fiscal year the awards were committed using annual appropriations that include new and competing renewal awards (type 1 and 2) and non-competing renewals (type 4 and 5). Unawarded commitments to pending out years of funding are not included. Awards were curated to match the award mechanisms of the BRAIN Circuits portfolio. For example,

- non-BRAIN R21/U21, R34/35/36/37, R61 to match the BRAIN Circuits Program exploratory projects (R21/U21, R34, 3-yr U01, R61)
- non-BRAIN R01, R15, DP1, DP2, DP5, U01, RF1/UF1 to match the BRAIN Circuits Program R01 and RF1
- non-BRAIN U19, P01, P50/56, RM1 to match the multi-component, research-center scale of the BRAIN Circuits Program U19s.

**Figure 3.**
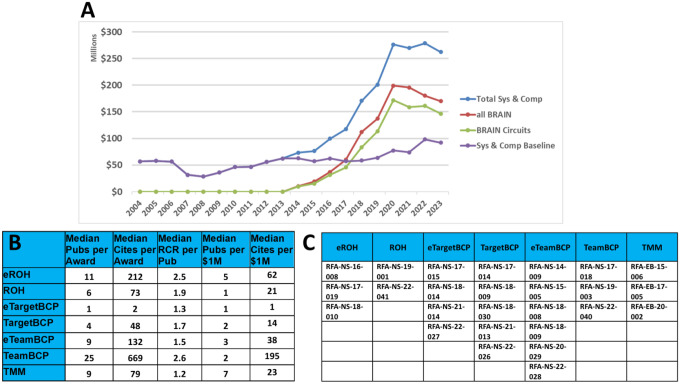
(A) NIH expenditures in systems and computational neuroscience for 10 years before and after launch of the BRAIN Initiative. Baseline is calculated from NIH RCDC (Research, Condition, and Disease Categorization) search of “neurosciences research” [OR] “computational neuroscience” (Sys & Comp NS), excluding BRAIN Awards. BRAIN Initiative awards are tallied as all Sys & Comp NS within BRAIN and all awards within the BRAIN Circuits funding announcements. (B) Performance measures for each funding mechanism, tabulating the median number of total publications per award, the median of total citations per award, and the median RCR among all publications within the funding category. Median publications per million and median cites per million were also calculated. (C) BRAIN Circuits Program Funding Announcements.

Similarly, all BRAIN Initiative awards by year in this categorization, and those issued specifically within the BRAIN Circuits Program, are plotted separately from selection by funding announcements (Figure 3C). The Total Systems and Computational Neuroscience curve is the sum of the neuroscience baseline plus the BRAIN expenditures by year. To manage annual BRAIN expenditures within annualized budget allocations, many of the BRAIN R01s were issued as RF1 where years 1–3 are funded in the initial award year and years 4–5 are awarded after an administrative review (type 4). The BRAIN curves rise for fiscal years 2014–2020 including both standard annual awards plus RF1 budgets for 3 years and flattens for fiscal years 2021–2023 due to currently unawarded, non-competing (type 4) continuations.

While the NIH baseline funding in this definition of neuroscience research grows relatively steadily by an average of 4.2% per year, the investments from the BRAIN Circuits Program climbs by an additional average of 12.5% per year. This creates a more than four-fold accumulation of investment. Since the NIH baseline includes both basic and disease-centered research and some neuroscience research outside the heksor concept, the BRAIN Circuits Program increment in funding for basic, disease-agnostic discovery in systems and computational neuroscience is substantially higher than 4X.

Figure 2B reports bibliometric productivity measures for each research track across all awards within each funding mechanism, including the median publications per award, the median citations per award and the median Relative Citation Ratio (RCR) per publication. The RCR is a normalized index of impact (Hutchins et al., 2016), which is a measure of citation relative to the topic neighborhood for the year of publication. An RCR of 1.0 represents a median citation count relative to that year and topic neighborhood.

The median RCR for these BRAIN programs, ranging 1.2 to 2.6, demonstrate high citation rates compared to median citation rates for comparable neuroscience topics. Table S4A shows that many of the top awards have RCR >> 10.0. By these publication and citation measures, all of these systems neuroscience programs featured impressive impacts. The financial efficiency of the BRAIN Circuits Programs ranged from median values up to 5 publications per $1M and up to 195 citations per $1M. The TeamBCP, eROH, and eTeam BCP programs present a particularly high return per dollar at 195, 62, and 38 citations/$M, respectively.

As a measure of the effectiveness of the peer-review advancement from exploratory to elaborated funding, for each of the tracks, we calculated the rate of advancement for the exploratory versions to the intended, larger-scale awards (See Bader et al, 2024 for exemplar methodology). The BRAIN R34 tract to BRAIN R01 was 13%, reflecting a low-cost, ‘high-risk/high-reward’ path to elaborated BRAIN programs. The advancement rate for the exploratory TeamBCP awards was a healthy 59% (Bader et al., 2024), and the rate of successful advancement of the exploratory ROH awards was an impressive 73%. When evaluating the advancement rate to any/all subsequent NIH awards, including other ICs and BRAIN, the respective successes rose to 30%, 76% and 73%.

For comparison, we similarly calculated the transition successes for other comparable NIH programs for the same range of fiscal years for the same award years as the comparable BRAIN funding mechanisms. The all-NIH advancement rate for

- ‘fundamental neuroscience’ (disease agnostic research) IC Program Projects (P01) was 85%, as a comparable multi-component program
- the NIMH Conte Centers was 73%, as a large, topic-focused neuroscience research center
- the CRCNS program as comparable combination of experimental and computational approaches was 95%

We suggest this reveals a strong continuity of meritorious work within research programs that foster collaborative effort, as measured by peer review. Of course, such transition success to subsequent awards is an incomplete measure of impact, as are bibliometric measures that are objectively quantitative but rely heavily on traditional academic publication/citation metrics. For example, one-shot, tool-building exercises might spawn large numbers of subsequent use in the research community but not advancement to a related new award or broad citation, which is particularly characteristic for the BRAIN Circuits Program exploratory R34 and TMM tool-building awards.

Whereas Figure 2 tabulates a snapshot of productivity and financial efficiency data for accumulated publications and citations for all awards at all stages of award - including first year of award where some would have few to zero publications out to accumulation of citations years past the end date of awards, Figure 3 shows the growth of publications, citations and RCR from the successive award years for each of the full-scale programs for each research track (TargetBCP, TeamBCP, ROH, and TMM). All of the programs accumulated publications and citations at a healthy rate of return, especially for the team-research BCP program. Publications from all the funding tracts posted RCR metrics well above 1.0 with good consistency.

For each research track, in Table S3A, we illustrate the breadth of topics with highest impact by noting a current snapshot of the highest performing 5 awards per track measured by RCR. A healthy spectrum of neuroscience research topics came from these investigator-initiated applications. Well-represented are sensation/perception; movement and complex behaviors, such as navigation; decision; social behaviors and behavioral states.

Similarly, as a snapshot of public impact, we turned to Altmetric scores within each research tract in Table S3B, which are a measure designed to identify how much media attention a research publication has received. The high popularity topics for the large and small Brain Circuits awards include interesting behaviors, such as sleep/wake, maternal behaviors and odor pleasantness; understanding structural details of the brain; and mechanisms of SARS-Co-2 infections on anosmia (Bader et al., 2025(. Top topics for the functional human neuroscience centered around neural decoding of speech/language and neural encoding for speech prostheses. The top topic of public interest for the TMM program involved identifying anxiety activity in mnemonic and brain hormonal axes. It is noteworthy how some of these projects awarded for their merit in fundamental neuroscience offer examples of manuscripts that attest to early translational impact, indicated by asterisks.

## Conclusions

The NIH BRAIN Initiative’s primary goal is to enable neuroscience broadly (Ngai, 2024). Its impact is recognized across the NIH neuroscience IC priorities (see ***BRAIN at 10*** messages of the 10 neuroscience Institute Directors in The BRAIN Blog). We offer that the BRAIN Initiative Circuits Program has substantially advanced and changed the landscape of research funding in fundamental, pre-translational discovery in systems and computational neuroscience. A qualitative testament to this conclusion is reflected in an informal poll of IC-contributing Program Directors that work within the BRAIN Circuits Program Team (Table S4). For objective measure, the raw and normalized citation indices offer impressive measures of scientific impact across all the funding tracts, and particularly high performance by the team-research TeamBCP program (Figure 3B).

In summary, the BRAIN Initiative has invested approximately $1B into fundamental, quantitative, systems neuroscience over 10 years (Figure 1B), reaching more than four-fold increase in the rate of funding in basic experimental neuroscience compared to the 10 years prior to the BRAIN Initiative (Figure 3A). Another important feature of the BRAIN Circuits Program was that a strategy of peer-reviewed, staged funding from exploratory/pilot projects to complex, hypothesis-driven research of high merit was highly effective. By measures of publication and citation rates, the BRAIN Circuits Program has produced fiscally-efficient, high-impact discovery in basic neuroscience research across a broad spectrum of research topics in behavioral, systems and computational neuroscience.

## METHODOLOGY

### Budgetary and Grant Data

BCP program budget and numbers of applications (FY2014–2022) were pulled from NIH FASTR and publications from iCITE. Number of publications, publication citations, average citations, and Relative Citation Rates were extracted from iCITE and custom Python scripts by the BRAIN Office of Budget.

### Landscape maps:

All of the notices of funding opportunities from the brain circuits portfolio (integrative and quantitative neuroscience) were queried in the NIH internal iSEARCH 3.0 portfolio analysis platform to yield all of the unique awards from 2014–2023. The titles and abstracts from the resulting competing awards were input into a Python script using Matplotlib library and Seaborn interface in order to derive the cluster visualizations from words that appeared frequently. The Python script was adapted from https://amueller.github.io/word_cloud/

### Continuity analysis

The BRAIN Circuits Program portfolio of awarded grants were downloaded using an NIH-internal database search engine (Query View Report, QVR) that can recapitulate the public, awarded grant information available from NIH RePORTER as linked in this report, including the keyword fingerprint field (Matchmaker). For each Principal Investigator (PI) for a project, grants across the NIH that shared similarity in scientific content to the original project (derived from the similarity fingerprint) were identified. Unawarded, non-research related mechanisms, and non-basic science related grants were filtered out. Manual review of the title and abstract were used to confirm that the selected project was a continuation of the original grant’s work by the original (Multi-)PI.

Continuity analyses were constructed for each of our BRAIN Circuits Programs with the general convention of smaller, exploratory awards leading to larger grants: U01→U19, R34→R01/U01, and eROH→full ROH. The ‘downstream’ projects that resulted from the original project included both BRAIN and non-BRAIN grants.

Comparison groups were determined by examining non-BRAIN NIH initiatives that had similar goals and funding/administrative structures as the BRAIN announcements. The same set of processes/business rules for continuity plotting were applied.

Success rate calculations within a funding track for the continuity analyses were performed using the following formulas: (Number of similar downstream awards)/(Number of original awards). Note that if multiple original awards led to a similar downstream award, that batch of awards were only counted once in the numerator, and that a given BRAIN award could spin off multiple, successful, downstream awards.

### GitHub Data:

Customized scripts using all of BRAIN Circuits Program application IDs were written to extract repositories from annual NIH Research Performance Progress Reports in conjunction with the number of stars, watchers, and forks associated with each repository.

## Supplementary Material

1

## Figures and Tables

**Figure 4.**
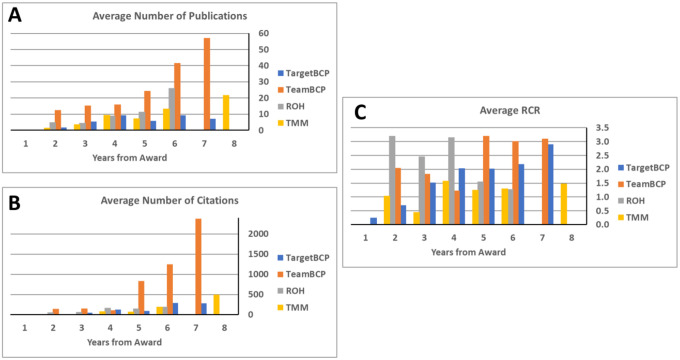
Accumulating performance measures for awards in the elaborated version of each funding tract by year from award. (A) Average number of publications for awards within each track. (B) Average number of citations for awards within each track. (C) Average RCR for publications within each award for each track.
