# Supplementary material for: The NIH BRAIN Initiative’s Impacts in Systems and Computational Neuroscience and Team-Scale Research, 2014–2023
Source: bioRxiv. 2025 May 20:2025.01.30.635684. Originally published 2025 Feb 1. Preprint. [Version 2] doi: 10.1101/2025.01.30.635684 (PMC11838304; doi:10.1101/2025.01.30.635684)
Supplement: 1 [file NIHPP2025.01.30.635684V2-supplement-1.pdf]

bioRxiv preprint doi: https://doi.org/10.1101/2025.01.30.635684; this version posted May 20, 2025. The copyright holder for this preprint (which was not certified by peer review) is the author/funder. This article is a US Government work. It is not subject to copyright under 17 USC 105 and is also made available for use under a CC0 license.

Supplemental Information

BRAIN Initiative Impacts in Systems and Computational Neuroscience and Team-Scale Research, 2014-2023

Farah Bader[2], Karen K. David[1,3], Hermon Gebrehiwet[1], Crystal Lantz[1], Grace C.Y. Peng[1,3], Mauricio Rangel-Gomez[1,3], James Gnadt[1,3,4]

bioRxiv preprint doi: https://doi.org/10.1101/2025.01.30.635684; this version posted May 20, 2025. The copyright holder for this preprint (which was not certified by peer review) is the author/funder. This article is a US Government work. It is not subject to copyright under 17 USC 105 and is also made available for use under a CC0 license.

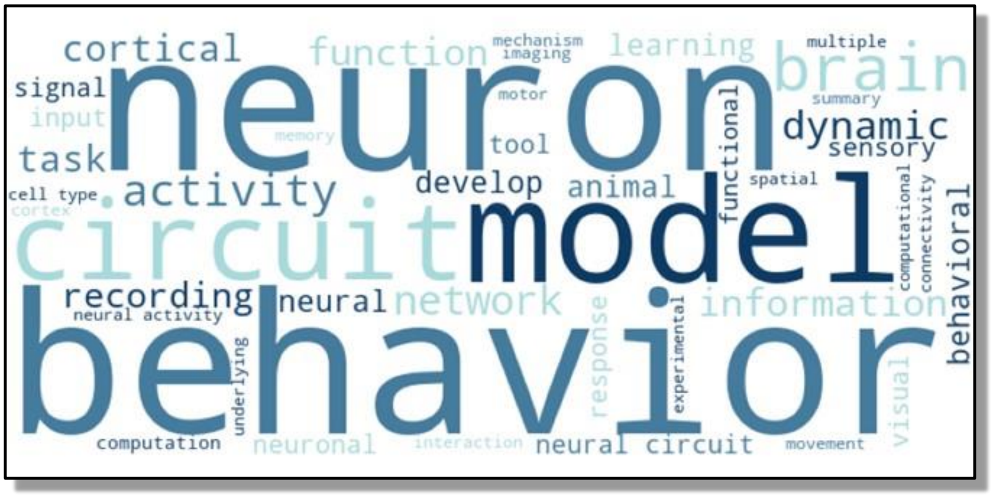

Table S1: Word Cloud from titles and abstracts of all BRAIN BCP awards. As designed, Behavioral, Systems and Computational Neuroscience are well represented.

bioRxiv preprint doi: https://doi.org/10.1101/2025.01.30.635684; this version posted May 20, 2025. The copyright holder for this preprint (which was not certified by peer review) is the author/funder. This article is a US Government work. It is not subject to copyright under 17 USC 105 and is also made available for use under a CC0 license.

A

Title	T or M or M	GitHub Stars
Next-Generation Calcium Imaging Analysis Methods	Method	1648
Human Neocortical Neurosolver	Model	474
Embedded Ensemble Encoding	Theory	256
Uncovering Population-Level Cellular Relationships to Behavior via Mesoscale Networks	Model	58
Data-driven analysis for neuronal dynamic modeling	Method	56
Methods from Computational Topology and Geometry for Analyzing Neuronal Tree and Graph Data	Method	55
Measuring, Modeling, and Modulating Cross-Frequency Coupling	Model	52
Modeling the structure-function relation in a reconstructed cortical tissue	Model	46
Real-time statistical algorithms for controlling neural dynamics and behavior	Method	33

B

Repository	Purpose	Stars	Watchers
https://github.com/flatironinstitute/CalmAn	Computational toolbox for large-scale Ca+2 imaging analysis	540	50
https://github.com/flatironinstitute/CalmAn-MATLAB	Matlab pipeline for large scale Calcium imaging analysis	236	60
https://github.com/zhoupc/CNMF_E	Constrained Non-negative Matrix Factorization for MicroEndoscopic data	128	53

Table S2 A: TMM projects with the highest number of cumulative GitHub Stars that produced highly popular computational tools in the form of normative theories, predictive models and computational algorithms. **B:** Highest rated GitHub repositories from TMM awards. These highly popular repositories are supported by multiple projects and programs. Snapshot from November 2024.

bioRxiv preprint doi: https://doi.org/10.1101/2025.01.30.635684; this version posted May 20, 2025. The copyright holder for this preprint (which was not certified by peer review) is the author/funder. This article is a US Government work. It is not subject to copyright under 17 USC 105 and is also made available for use under a CC0 license.

PMID	Manuscript Title	Altmetric
**TargetBCP R01**		
31473062	A Rare Mutation of β1-Adrenergic Receptor Affects Sleep/Wake Behaviors.	1380
37425937	Neuronal wiring diagram of an adult brain.	1072
36007021	A cognitive process occurring during sleep is revealed by rapid eye movements.	1029
32939091	Deep posteromedial cortical rhythm in dissociation.	801
31619542	Mutant neuropeptide S receptor reduces sleep duration with preserved memory consolidation.	775
31150625	Human Gut Microbiota from Autism Spectrum Disorder Promote Behavioral Symptoms in Mice.*	775
33057202	PIEZO2 in sensory neurons and urothelial cells coordinates urination.	695
31642810	An Integrated Brain-Machine Interface Platform With Thousands of Channels.	628
38092914	A transcriptomic taxonomy of mouse brain-wide spinal projecting neurons.	600
**TeamBCP U19**		
32937591	Non-neuronal expression of SARS-CoV-2 entry genes in the olfactory system suggests mechanisms underlying COVID-19-associated anosmia.*	2777
36603070	A mesothelium divides the subarachnoid space into functional compartments.	2215
37730989	Neural circuitry for maternal oxytocin release induced by infant cries.	2095
35381183	The perception of odor pleasantness is shared across cultures.	1419
28445462	Cell diversity and network dynamics in photosensitive human brain organoids.*	891
34381215	Oxytocin neurons enable social transmission of maternal behavior.	824
32554567	Manipulating synthetic optogenetic odors reveals the coding logic of olfactory perception	784
37286598	Antagonistic circuits mediating infanticide and maternal care in female mice.	723
34930847	De novo mutations in childhood cases of sudden unexplained death that disrupt intracellular Ca2+ regulation.*	721
36889318	Mouse spontaneous behavior reflects individual variation rather than estrous state.	694

bioRxiv preprint doi: https://doi.org/10.1101/2025.01.30.635684; this version posted May 20, 2025. The copyright holder for this preprint (which was not certified by peer review) is the author/funder. This article is a US Government work. It is not subject to copyright under 17 USC 105 and is also made available for use under a CC0 license.

36572698	Multimodal monitoring of human cortical organoids implanted in mice reveal functional connection with visual cortex.*	654
34381214	A metabolic function of the hippocampal sharp wave-ripple	612
**ROH U01**		
31019317	Speech synthesis from neural decoding of spoken sentences.	3065
34260835	Neuroprosthesis for Decoding Speech in a Paralyzed Person with Anarthria.	2705
32231340	Machine translation of cortical activity to text with an encoder-decoder framework.	1357
32375031	Replay of Learned Neural Firing Sequences during Rest in Human Motor Cortex.	772
33462446	State-dependent responses to intracranial brain stimulation in a patient with depression.*	645
35511978	The geometry of domain-general performance monitoring in the human medial frontal cortex.	614
29779940	Encoding of Articulatory Kinematic Trajectories in Human Speech Sensorimotor Cortex.	609
**TMM R01**		
29397273	Anxiety Cells in a Hippocampal-Hypothalamic Circuit.	943

Table S3: Publications (PubMed Identifier, PMID), per research track, from awards that have Altmetric scores above 600. * Manuscripts that attest to early translational impact

bioRxiv preprint doi: https://doi.org/10.1101/2025.01.30.635684; this version posted May 20, 2025. The copyright holder for this preprint (which was not certified by peer review) is the author/funder. This article is a US Government work. It is not subject to copyright under 17 USC 105 and is also made available for use under a CC0 license.

Table S4. Sample quotations from an informal poll of IC-contributing Program Directors that work within the BRAIN Circuits Program Team.

What phrase would you use to describe [the BRAIN Circuits Program] science?

- "Ambitious projects that integrate the interests of multiple BRAIN ICs."
- "Transformative, multidisciplinary studies of neural circuits and behavior."
- "Capturing the brain in action."
- "High-risk high-reward collaborative Systems & Computational Neuroscience"
- "Putting it all together, from biology to behavior."
- "Linking neural circuit dynamics with behavior."
- "The Science of Complex Neural and Behavioral Function"

How has the science stimulated and supported by [the BRAIN Circuits Program] advanced the science of your IC?

- "[It has stimulated] Team science involving identified cell types and computational modeling."
- In particular [the BRAIN Circuits Program] endeavors that supported studies to optimize large scale recordings and modulation of the CNS technologies and Targeted Brain Circuits BCP are beginning to pave the way for a more detailed examination of mental health relevant behaviors."
- "The TargetedBCP program . . . have produced some interesting research that gets at the heart of NINDS mission: 'to seek fundamental knowledge about the brain and nervous system'."
- "The NIMH human basic neuroscience portfolio has benefitted from BRAIN investments in ROH [Research Opportunities in Humans]. The number of NIMH applications proposing studies of human neurosurgical patients has increased dramatically over the last 10 years (from near zero to multiple applications each council round)."
- "Many of the [the BRAIN Circuits Program] applications have developed new tools and sensors that are being leveraged in a variety of projects in basic behavior and neuroscience at NIMH that examine neural circuit function in behaving animals."
- "Three main 'footprints' right now - 1) more integration of computational approaches integrated into behavioral and cognitive neuroscience' projects . . . a big advance over the behavioral pharmacologic approaches still common in NIDA grants. 2) increases in 'multimodal' / multidimensional and higher-resolution measurement of behavior, even in the context of straightforward, task-driven cognitive neuroscience proposals, 3) more consideration of ecologic

bioRxiv preprint doi: https://doi.org/10.1101/2025.01.30.635684; this version posted May 20, 2025. The copyright holder for this preprint (which was not certified by peer review) is the author/funder. This article is a US Government work. It is not subject to copyright under 17 USC 105 and is also made available for use under a CC0 license.

validity and use of technology to test neurobehavioral processes in naturalistic environments."

Briefly describe the science you see as the highest priority areas/approaches to be put forward by [the BRAIN Circuits Program]

- "The themes cut across IC priorities. If we don't follow up on the last decade of investments in [BRAIN Initiative] portfolios, we lose out on much of the momentum built on both those fronts. The brain does not operate according to IC priorities, and [the BRAIN Circuits] portfolio offers the unique opportunity to not artificially silo the brain and instead study it as a whole."
- "To provide support to collaborative efforts between theoretical and experimental neuroscientists to leverage the latest advances in the field of AI to generate testable hypotheses about how the brain circuits process incoming information and how at a circuit level the activity of the brain gives rise to behavior . . .."
- "Computational modeling approaches that DO NOT use AI."
- "Compared to significant strides made in understanding neural circuits function in relation to complex behaviors, studies examining mechanistic relationship between developing brain circuits and behaviors are lagging . . . Encouraging this type of mechanistic, normative developmental studies would allow researchers to identify how deviations in neurodevelopmental trajectories of neural circuits might be linked to maladaptive behaviors observed across a range of brain disorders."
- "I am worried about . . . support for systems neuroscience, especially for higher budget, collaborative cutting-edge projects that involve more risk."
- "Cell-specific neurophysiology techniques . . . need BRAIN support to develop."
- "Integrated approaches to understanding functional neural circuits with cell-type specificity. This area of research builds on molecular tools that have been developed (in part) by BRAIN and will provide the necessary foundation (and potential targets) for the efforts proposed under the Precision Molecular Circuit Therapies Domain."
- "Computational models and analytical approaches for understanding high density sampling of neural signals. Technological advances in data acquisition systems . . . have revolutionized neurophysiology. However, there is a need for next generation models and analytics that can interpret neural signals collected simultaneously across multiple brain regions to provide a better understanding of the real-time neural system dynamics that support cognitive, affective, social, and motor behaviors. These approaches, and the knowledge gained from them, will be critical for the success of closed-loop neuromodulatory systems and therapies."

bioRxiv preprint doi: https://doi.org/10.1101/2025.01.30.635684; this version posted May 20, 2025. The copyright holder for this preprint (which was not certified by peer review) is the author/funder. This article is a US Government work. It is not subject to copyright under 17 USC 105 and is also made available for use under a CC0 license.

- “General theoretical frameworks of neural circuit function and behavior that consider the circular relationship between perceptual input and behavioral output in the context of control systems . . ..”
- Further expansion of the BBQS program to include additional measures of sensory perception from the animal's perspective. Development of tools that enable more precise measures of motor output (e.g., flexible electrodes in muscles) that are used alongside continues measures of behavior and neural activity . . ..”
- “Within these goals we prioritize comparative approaches and advancement of computational models that are a synthesis of theory-guided (supervised) and data-driven approaches.
- “We need to continue a [TeamBCP] with greater emphasis on formal/computational modeling and comparative thinking.”
- “[Investigations] providing rationales that link circuit function across evolution and species, even if they are studying only one species in a project.”
- “I see a need for continued, growing support and requirement of use of computational approaches.”
