# Supplementary material for: The NIH BRAIN Initiative’s Impacts in Systems and Computational Neuroscience, 2014–2023
Source: bioRxiv. 2025 Feb 1:2025.01.30.635684. Preprint. [Version 1] doi: 10.1101/2025.01.30.635684 (PMC11838304; doi:10.1101/2025.01.30.635684)
Supplement: Supplementary file 1 [file NIHPP2025.01.30.635684V1-supplement-1.pdf]

bioRxiv preprint doi: https://doi.org/10.1101/2025.01.30.635684; this version posted February 1, 2025. The copyright holder for this preprint (which was not certified by peer review) is the author/funder. This article is a US Government work. It is not subject to copyright under 17 USC 105 and is also made available for use under a CC0 license.

Supplemental Information

BRAIN Initiative Impacts in Systems and Computational Neuroscience, 2014-2023

Farah Bader[2], Karen K. David[1,3], Hermon Gebrehiwet[1], Crystal Lantz[1], Grace C.Y. Peng[1,3], Mauricio Rangel-Gomez[1,3], James Gnadt[1,3,4]

bioRxiv preprint doi: https://doi.org/10.1101/2025.01.30.635684; this version posted February 1, 2025. The copyright holder for this preprint (which was not certified by peer review) is the author/funder. This article is a US Government work. It is not subject to copyright under 17 USC 105 and is also made available for use under a CC0 license.

Appendix:

BRAIN Initiative, Integrative and Quantitative Approaches Team (past and current):

Team Co-Leads: Karen David (NINDS; 2024), Jim Gnadt* (NINDS; 2013-2024), Grace Peng* (NIBIB; 2014-2022; Team C Co-Lead, 2013), Mauricio Rangel-Gomez (NIMH; 2022-2024)

Team Members: Afomeya Agonafer (NCCIH), Farah Bader (NINDS), Olivier Berton (NIDA), Andrew Breeden (NIMH), Claudette Brooks (ORWH), Bettina Buhring* (NIMH), Janet Cyr (NIDCD), Karen David (NINDS), Hsiao Yu (Christina) Fang (NINDS), Fernando Fernandez (NIMH), Michele Ferrante (NIMH), Martha Flanders (NEI), Hermon Gebrehiwet (NINDS), Marie Gill (NINDS), Tom Greenwell (NEI), Sofiya Hupalo (NIMH), Douglas Kim (NIMH), Eunyoung Kim (NIMH), Crystal Lantz (NINDS), John Matochik (NIAAA), Heidi Matos Galicia (NINDS), Brett Miller (NICHD), Pantea Moghimi (NINDS), Jessica Mollick (NIDA), Joe Monaco (NINDS), Holly Moore (NIDA), Antonio Noronha* (NIAAA), Leslie Osborne (NINDS), Christopher Platt* (NIDCD), Amy Poremba (NIDCD), Elizabeth Powell (NIAAA), Erin Quinlan (NCCIH), Tom Radman (NIDA), Andrew Rossi (NIMH), Merav Sabri (NIDCD), Dana Schloesser (OBSSR), David Shurtleff* (NCCIH), Janine Simmons (OBSSR), Coryse St Hillaire-Clarke (NIA), Michael Steinmetz* (NEI), Luke Stoeckel (NIA), Ned Talley* (NINDS), Natalie Trzcinski (NINDS), Aleksandra Vicentic (NIMH), Susan Volman* (NIDA), Molly Wagster* (NIA), Charles Wright (NEI), Susan Wright (NIDA)

Scientific Review Officers: Jasenka Borzan (CSR), Robert Elliot (CSR), Nick Gaiano (CSR), Dennis Hlasta (NIBIB), Li Jia (NINDS), Ernie Lyons (NINDS), Marilyn Moore-Hoon (NINDS), Matthew Oh (CSR), Tania Pasternak (NINDS), Raul Saavedra (NINDS), Kadharbatcha Saleem (CSR), Joel Saydoff (NINDS), Natalia Strunnikova (NINDS), Kirk Thompson (CSR), Delany Torres (NINDS)

***Founding members** (originally combined with technology-building Team B; Richard Conroy (NIBIB), Changhai Cui (NIAAA), Jim Gnadt (NINDS; Team E Co-Lead), Bill Heetderks (NIBIB), Kip Ludwick (NINDS; Team B Co-Lead), Mac Mackiewicz (NIA), Roger Sorenson (NIAAA), Ned Talley* (NINDS; Co-Chair of BRAIN Coordinating Team), Liz Webber (NINDS)

bioRxiv preprint doi: https://doi.org/10.1101/2025.01.30.635684; this version posted February 1, 2025. The copyright holder for this preprint (which was not certified by peer review) is the author/funder. This article is a US Government work. It is not subject to copyright under 17 USC 105 and is also made available for use under a CC0 license.

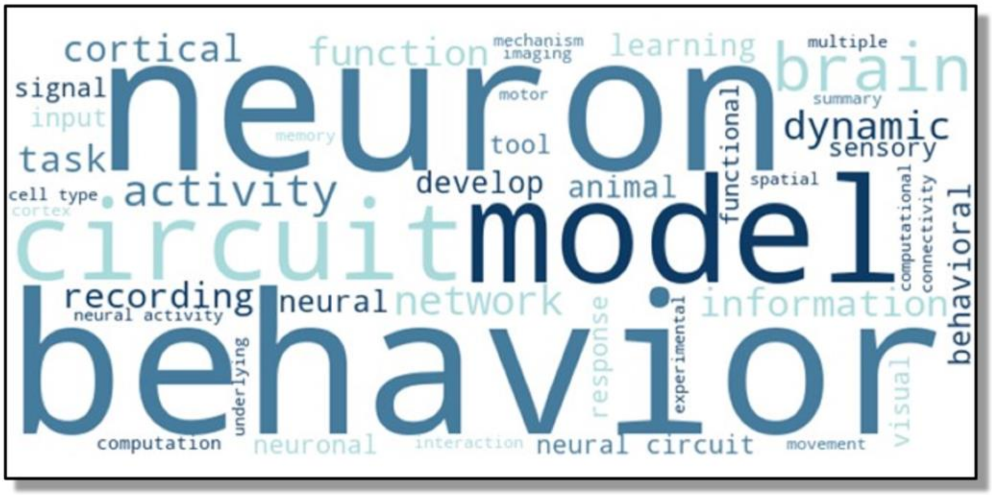

Table S1A: Word Cloud from titles and abstracts of all BRAIN BCP awards. Behavioral, Systems and Computational Neuroscience are well represented.

eROH	ROH	eTargetBCP	TargetBCP	eTeamBCP	TeamBCP	TMM
RFA-NS-16-008	RFA-NS-19-001	RFA-NS-17-015	RFA-NS-17-014	RFA-NS-14-009	RFA-NS-17-018	RFA-EB-15-006
RFA-NS-17-019	RFA-NS-22-041	RFA-NS-18-014	RFA-NS-18-009	RFA-NS-15-005	RFA-NS-19-003	RFA-EB-17-005
RFA-NS-18-010		RFA-NS-21-014	RFA-NS-18-030	RFA-NS-18-008	RFA-NS-22-040	RFA-EB-20-002
		RFA-NS-22-027	RFA-NS-21-013	RFA-NS-18-029		
			RFA-NS-22-026	RFA-NS-20-029		
				RFA-NS-22-028		

bioRxiv preprint doi: https://doi.org/10.1101/2025.01.30.635684; this version posted February 1, 2025. The copyright holder for this preprint (which was not certified by peer review) is the author/funder. This article is a US Government work. It is not subject to copyright under 17 USC 105 and is also made available for use under a CC0 license.

Title	T or M or M	GitHub Stars
Next-Generation Calcium Imaging Analysis Methods	method	1648
Human Neocortical Neurosolver	model	474
Embedded Ensemble Encoding	theory	256
Uncovering Population-Level Cellular Relationships to Behavior via Mesoscale Networks	model	58
Data-driven analysis for neuronal dynamic modeling	method	56
Methods from Computational Topology and Geometry for Analyzing Neuronal Tree and Graph Data	method	55
Measuring, Modeling, and Modulating Cross-Frequency Coupling	model	52
Modeling the structure-function relation in a reconstructed cortical tissue	model	46
Real-time statistical algorithms for controlling neural dynamics and behavior	method	33

Table S2A: TMM projects that produced highly popular computational tools in the form of normative theories, predictive models and computational algorithms.

**Repository**	**Purpose**	**Stars**	**Watchers**
https://github.com/flatironinstitute/CaImAn	Computational toolbox for large-scale Ca+2 imaging analysis	540	50
https://github.com/flatironinstitute/CaImAn-MATLAB	Matlab pipeline for large scale Calcium imaging analysis	236	60
https://github.com/zhoupc/CNMF_E	Constrained Non-negative Matrix Factorization for MicroEndoscopic data	128	53

Table S2B: These highly popular repositories are supported by multiple projects and programs by the same Principal Investigator (L.P. Paninski) who has had two TMM awards and a Team BCP award.

bioRxiv preprint doi: https://doi.org/10.1101/2025.01.30.635684; this version posted February 1, 2025. The copyright holder for this preprint (which was not certified by peer review) is the author/funder. This article is a US Government work. It is not subject to copyright under 17 USC 105 and is also made available for use under a CC0 license.

bioRxiv preprint doi: https://doi.org/10.1101/2025.01.30.635684; this version posted February 1, 2025. The copyright holder for this preprint (which was not certified by peer review) is the author/funder. This article is a US Government work. It is not subject to copyright under 17 USC 105 and is also made available for use under a CC0 license.

Grant Number	Grant Title	RCR
**TargetBCP R01**		
R01NS104944	Multiplex imaging of neuronal activity and signaling dynamics underlying learning in discrete amygdala circuits of behaving mice	74.0
R01NS109990	Neuromodulation of Brain States	67.4
R01NS104899	Dissecting Sensorimotor Pathways Underlying Social Interactions: Models, Circuits, and Behavior	54.3
R01NS104925	The self-tuning brain: cellular and circuit mechanisms of behavior	54.3
R01NS108410	Studying perceptual decision-making across cortex by combining population imaging, connectomics, and computational modeling	46.8
**TeamBCP U19**		
U19NS104590	Towards a Complete Description of the Circuitry Underlying Sharp Wave-Mediated Memory Replay	160.6
U19NS104649	Computational and circuit mechanisms underlying motor control	121.3
U19NS104653	Sensorimotor processing, decision making, and internal states: towards a realistic multiscale circuit model of the larval zebrafish brain	113.6
U19NS112953	Cracking the Olfactory Code	109.1
U19NS104648	Mechanisms of neural circuit dynamics in working memory and decision-making	94.8
**ROH U01**		
U01NS117839	Neuronal mechanisms of human episodic memory	22.1
U01NS117838	Neurostimulation and Recording of Real World Spatial Navigation in Human	19.2
U01NS121616	An integrated single-neuronal, population-, local network- and stimulation-based prefrontal investigation of human social cognition	13.5
U01NS117765	The neural coding of speech across human languages	13.3
U01NS121471	Computational Neuroscience of Language Processing in the Human Brain	6.8
**TMM R01**		
R01EB022880	Diagnosis of Alzheimers Disease Using Dynamic High-Order Brain Networks	166.4
R01EB022913	Next-Generation Calcium Imaging Analysis Methods	90.9
R01EB022717	Multimodal modeling framework for fusing structural and functional connectome data	50.8
R01EB022907	Novel Bayesian linear dynamical systems-based methods for discovering human brain circuit dynamics in health and disease	39.9
R01EB022720	Graph theoretical analysis of the effect of brain tumors on functional MRI networks	38.1

Table S3A: Highest impact awards by funding track as measured by RCR.

bioRxiv preprint doi: https://doi.org/10.1101/2025.01.30.635684; this version posted February 1, 2025. The copyright holder for this preprint (which was not certified by peer review) is the author/funder. This article is a US Government work. It is not subject to copyright under 17 USC 105 and is also made available for use under a CC0 license.

PMID	Manuscript Title	Altmetric
**TargetBCP R01**		
31473062	A Rare Mutation of β1-Adrenergic Receptor Affects Sleep/Wake Behaviors.	1380
37425937	Neuronal wiring diagram of an adult brain.	1072
36007021	A cognitive process occurring during sleep is revealed by rapid eye movements.	1029
32939091	Deep posteromedial cortical rhythm in dissociation.	801
31619542	Mutant neuropeptide S receptor reduces sleep duration with preserved memory consolidation.	775
31150625	Human Gut Microbiota from Autism Spectrum Disorder Promote Behavioral Symptoms in Mice.*	775
33057202	PIEZO2 in sensory neurons and urothelial cells coordinates urination.	695
31642810	An Integrated Brain-Machine Interface Platform With Thousands of Channels.	628
38092914	A transcriptomic taxonomy of mouse brain-wide spinal projecting neurons.	600
**TeamBCP U19**		
32937591	Non-neuronal expression of SARS-CoV-2 entry genes in the olfactory system suggests mechanisms underlying COVID-19-associated anosmia.*	2777
36603070	A mesothelium divides the subarachnoid space into functional compartments.	2215
37730989	Neural circuitry for maternal oxytocin release induced by infant cries.	2095
35381183	The perception of odor pleasantness is shared across cultures.	1419
28445462	Cell diversity and network dynamics in photosensitive human brain organoids.*	891
34381215	Oxytocin neurons enable social transmission of maternal behaviour.	824
32554567	Manipulating synthetic optogenetic odors reveals the coding logic of olfactory perception	784
37286598	Antagonistic circuits mediating infanticide and maternal care in female mice.	723
34930847	De novo mutations in childhood cases of sudden unexplained death that disrupt intracellular Ca2+ regulation.*	721
36889318	Mouse spontaneous behavior reflects individual variation rather than estrous state.	694
36572698	Multimodal monitoring of human cortical organoids implanted in mice reveal functional connection with visual cortex.*	654
34381214	A metabolic function of the hippocampal sharp wave-	612

bioRxiv preprint doi: https://doi.org/10.1101/2025.01.30.635684; this version posted February 1, 2025. The copyright holder for this preprint (which was not certified by peer review) is the author/funder. This article is a US Government work. It is not subject to copyright under 17 USC 105 and is also made available for use under a CC0 license.

	ripple	
**ROH U01**		
31019317	Speech synthesis from neural decoding of spoken sentences.	3065
34260835	Neuroprosthesis for Decoding Speech in a Paralyzed Person with Anarthria.	2705
32231340	Machine translation of cortical activity to text with an encoder-decoder framework.	1357
32375031	Replay of Learned Neural Firing Sequences during Rest in Human Motor Cortex.	772
33462446	State-dependent responses to intracranial brain stimulation in a patient with depression.*	645
35511978	The geometry of domain-general performance monitoring in the human medial frontal cortex.	614
29779940	Encoding of Articulatory Kinematic Trajectories in Human Speech Sensorimotor Cortex.	609
**TMM R01**		
29397273	Anxiety Cells in a Hippocampal-Hypothalamic Circuit.	943

Table S3B: Table S4B lists all publications (PubMed Identifier, PMID) from awards that have Altmetric scores above 600. * Manuscripts that attest to early translational impact

bioRxiv preprint doi: https://doi.org/10.1101/2025.01.30.635684; this version posted February 1, 2025. The copyright holder for this preprint (which was not certified by peer review) is the author/funder. This article is a US Government work. It is not subject to copyright under 17 USC 105 and is also made available for use under a CC0 license.

Table S4. Sample quotations from an informal poll of IC-contributing Program Directors that work within the BRAIN Circuits Program Team.

What phrase would you use to describe [the BRAIN Circuits Program] science?

- "Ambitious projects that integrate the interests of multiple BRAIN ICs."
- "Transformative, multidisciplinary studies of neural circuits and behavior."
- "Capturing the brain in action."
- "High-risk high-reward collaborative Systems & Computational Neuroscience"
- "Putting it all together, from biology to behavior."
- "Linking neural circuit dynamics with behavior."
- "The Science of Complex Neural and Behavioral Function"

How has the science stimulated and supported by [the BRAIN Circuits Program] advanced the science of your IC?

- "[It has stimulated] Team science involving identified cell types and computational modeling."
- In particular [the BRAIN Circuits Program] endeavors that supported studies to optimize large scale recordings and modulation of the CNS technologies and Targeted Brain Circuits BCP are beginning to pave the way for a more detailed examination of mental health relevant behaviors."
- "The TargetedBCP program . . . have produced some interesting research that gets at the heart of NINDS mission: 'to seek fundamental knowledge about the brain and nervous system'."
- "The NIMH human basic neuroscience portfolio has benefitted from BRAIN investments in ROH [Research Opportunities in Humans]. The number of NIMH applications proposing studies of human neurosurgical patients has increased dramatically over the last 10 years (from near zero to multiple applications each council round)."
- "Many of the [the BRAIN Circuits Program] applications have developed new tools and sensors that are being leveraged in a variety of projects in basic behavior and neuroscience at NIMH that examine neural circuit function in behaving animals."
- "Three main 'footprints' right now - 1) more integration of computational approaches integrated into behavioral and cognitive neuroscience' projects . . . a big advance over the behavioral pharmacologic approaches still common in NIDA grants. 2) increases in 'multimodal' / multidimensional and higher-resolution measurement of behavior, even in the context of straightforward, task-driven cognitive neuroscience proposals, 3) more consideration of ecologic

bioRxiv preprint doi: https://doi.org/10.1101/2025.01.30.635684; this version posted February 1, 2025. The copyright holder for this preprint (which was not certified by peer review) is the author/funder. This article is a US Government work. It is not subject to copyright under 17 USC 105 and is also made available for use under a CC0 license.

validity and use of technology to test neurobehavioral processes in naturalistic environments."

Briefly describe the science you see as the highest priority areas/approaches to be put forward by [the BRAIN Circuits Program]

- "The themes cut across IC priorities. If we don't follow up on the last decade of investments in [BRAIN Initiative] portfolios, we lose out on much of the momentum built on both those fronts. The brain does not operate according to IC priorities, and [the BRAIN Circuits] portfolio offers the unique opportunity to not artificially silo the brain and instead study it as a whole."
- "To provide support to collaborative efforts between theoretical and experimental neuroscientists to leverage the latest advances in the field of AI to generate testable hypotheses about how the brain circuits process incoming information and how at a circuit level the activity of the brain gives rise to behavior . . . ."
- "Computational modeling approaches that DO NOT use AI."
- "Compared to significant strides made in understanding neural circuits function in relation to complex behaviors, studies examining mechanistic relationship between developing brain circuits and behaviors are lagging . . . Encouraging this type of mechanistic, normative developmental studies would allow researchers to identify how deviations in neurodevelopmental trajectories of neural circuits might be linked to maladaptive behaviors observed across a range of brain disorders."
- "I am worried about . . . support for systems neuroscience, especially for higher budget, collaborative cutting-edge projects that involve more risk."
- "Cell-specific neurophysiology techniques . . . need BRAIN support to develop."
- "Integrated approaches to understanding functional neural circuits with cell-type specificity. This area of research builds on molecular tools that have been developed (in part) by BRAIN and will provide the necessary foundation (and potential targets) for the efforts proposed under the Precision Molecular Circuit Therapies Domain."
- "Computational models and analytical approaches for understanding high density sampling of neural signals. Technological advances in data acquisition systems . . . have revolutionized neurophysiology. However, there is a need for next generation models and analytics that can interpret neural signals collected simultaneously across multiple brain regions to provide a better understanding of the real-time neural system dynamics that support cognitive, affective, social, and motor behaviors. These approaches, and the knowledge gained from them, will be critical for the success of closed-loop neuromodulatory systems and therapies."

bioRxiv preprint doi: https://doi.org/10.1101/2025.01.30.635684; this version posted February 1, 2025. The copyright holder for this preprint (which was not certified by peer review) is the author/funder. This article is a US Government work. It is not subject to copyright under 17 USC 105 and is also made available for use under a CC0 license.

- "General theoretical frameworks of neural circuit function and behavior that consider the circular relationship between perceptual input and behavioral output in the context of control systems . . .."
- Further expansion of the BBQS program to include additional measures of sensory perception from the animal's perspective. Development of tools that enable more precise measures of motor output (e.g., flexible electrodes in muscles) that are used alongside continues measures of behavior and neural activity . . .."
- "Within these goals we prioritize comparative approaches and advancement of computational models that are a synthesis of theory-guided (supervised) and data-driven approaches.
- "We need to continue a [TeamBCP] with greater emphasis on formal/computational modeling and comparative thinking."
- "[Investigations] providing rationales that link circuit function across evolution and species, even if they are studying only one species in a project."
- "I see a need for continued, growing support and requirement of use of computational approaches."
